# Fabrication of Highly Textured 2D SnSe Layers with Tunable Electronic Properties for Hydrogen Evolution

**DOI:** 10.3390/molecules26113319

**Published:** 2021-06-01

**Authors:** Qianyu Zhou, Mengya Wang, Yong Li, Yanfang Liu, Yuanfu Chen, Qi Wu, Shifeng Wang

**Affiliations:** 1 Department of Physics, and Innovation center of Materials for Energy and Environment Technologies, College of Science, Tibet University, Lhasa 850000, China; zhouqianyu@utibet.edu.cn (Q.Z.); wmy1430140113@sina.com (M.W.); xzuliyong@utibet.edu.cn (Y.L.); liuyanfang@utibet.edu.cn (Y.L.); 2 Institute of Oxygen Supply, Center of Tibetan Studies (Everest Research Institute), Tibet University, Lhasa 850000, China; 3 Key Laboratory of Cosmic Rays (Tibet University), Ministry of Education, Lhasa 850000, China; 4 School of Electronic Science and Engineering, and State Key Laboratory of Electronic Thin Films and Integrated Devices, University of Electronic Science and Technology of China, Chengdu 610054, China

**Keywords:** SnSe, 2D materials, hydrogen evolution, water splitting, DFT calculations, defect engineering

## Abstract

Hydrogen is regarded to be one of the most promising renewable and clean energy sources. Finding a highly efficient and cost-effective catalyst to generate hydrogen via water splitting has become a research hotspot. Two-dimensional materials with exotic structural and electronic properties have been considered as economical alternatives. In this work, 2D SnSe films with high quality of crystallinity were grown on a mica substrate via molecular beam epitaxy. The electronic property of the prepared SnSe thin films can be easily and accurately tuned in situ by three orders of magnitude through the controllable compensation of Sn atoms. The prepared film normally exhibited p-type conduction due to the deficiency of Sn in the film during its growth. First-principle calculations explained that Sn vacancies can introduce additional reactive sites for the hydrogen evolution reaction (HER) and enhance the HER performance by accelerating electron migration and promoting continuous hydrogen generation, which was mirrored by the reduced Gibbs free energy by a factor of 2.3 as compared with the pure SnSe film. The results pave the way for synthesized 2D SnSe thin films in the applications of hydrogen production.

## 1. Introduction

With the increase in CO_2_ emissions due to the use of traditional energy sources such as coal-fired electricity and oil-powered cars, the global warming leading to the sea level rise, glaciers melting, and frequent extreme weather has become an issue of increasing concern all over the world. To address this challenge, the concept of “carbon neutrality” was coined in 2005 [1,2], which referred to an equilibrium between the CO_2_ emissions in the atmosphere and the removal or capture of CO_2_ from the atmosphere generating net zero emissions. Several effective strategies have been proposed to reduce the amount of CO_2_ emissions, such as planting more trees, encouraging use of renewable energy sources, improving energy efficiency, and developing clean transportation. Hydrogen energy is such a kind of lean energy source with high efficiency and environmental benefits, whose “fuel” is hydrogen and/or hydrogen-containing compounds. Water splitting is considered to be the most promising pathway for hydrogen production, as it is recyclable, clean, and abundant. According to the thermodynamics of water splitting, the energy required to break one mole of water is 237 kJ [3,4], and the potential needed is at least 1.23 V [5,6]. To dissociate water molecules into hydrogen and oxygen by means of photoelectrochemical catalysis, various semiconductor materials have been used as catalysts, such as TiO_2_ [7,8], SrTiO_3_ [9,10], BiVO_4_ [10,11,12], and chalcogenide compounds [13,14].

To match the hydrogen reduction potential and water oxidation potential, wide-bandgap semiconductors are usually used as catalysts, which absorb only the sunlight in the UV range that comprises only a small amount of the solar irradiation energy. Other factors limiting the splitting efficiency include low charge separation efficiency, fast electron–hole recombination, and slow kinetics of the water redox reaction [4]. Although heterogenous junctions and Z-scheme structures have been designed to boost water splitting efficiency [10,15,16,17,18], most of the conventional catalysts still exhibit insufficiency because of the long migration path of the photogenerated carriers and lack of reactive sites.

Two-dimensional (2D) materials have attracted great interest in catalytic applications due to their anisotropic physical and electronic properties, high carrier mobility, tunable energy bandgaps, and high surface-to-bulk ratio facilitating enrichment of reactive sites and shortening the migration distance of carriers. Very recently, various 2D materials, such as graphene [19], graphitic carbon nitride (g-C_3_N_4_) [20], MoS_2_ [21], MoSe_2_ [22], WSe_2_ [23], and oxosulfide [24], have been synthesized for the oxygen evolution reaction (OER) and hydrogen evolution reaction (HER) activities. Belonging to the family of 2D transition metal chalcogenides, tin monoselenide (SnSe) has also received researchers’ attention due to its simplicity in structure and fabrication, inexpensiveness of constituent sources, superior performance for catalytic activity, and compatibility with diverse thin film preparation techniques. It has been studied as a catalyst for CO_2_ reduction [25]. However, there are few studies on the hydrogen generation using SnSe as the catalyst. SnSe crystalizes in an orthorhombic unit cell, in which atoms are strongly connected by covalent bonds within the layer whereas weak van der Waals interactions occur between the layers [26,27]. This unique structure enables SnSe to easily achieve 2D/2D or 2D/3D stacking forming heterostructures for the catalytic activity.

In this paper, layered SnSe films with high quality of crystallinity were successfully grown on mica substrates. A full width at half maximum (FWHM) of the XRD rocking curve was achieved as narrow as 0.121° on the SnSe (004) plane, which might be the best value ever reported and suggests highly textured growth orientation along its c-axis with excellent crystallization quality. Electrical measurements revealed that the films showed a p-type conductivity due to Sn vacancies in the film with a carrier mobility as high as 34 cm^2^/(Vs) and a sheet resistivity of 1.5 × 10^4^ Ω/square. The vacant defects can be effectively tuned by a separate elemental tin compensation source, tailoring resistivity in the range of three orders of magnitude. First-principle calculations using the Vienna Ab Initio Simulation Package (VASP) revealed that the presence of Sn vacancies in the SnSe film reduced the Gibbs free energy by a factor of 2.3 as compared with the pure SnSe. It was explained that Sn vacancies on the surface of layers provide more reactive sites and favor separation and transportation of photogenerated electrons, facilitating the continuous hydrogen evolution reaction. Our results pave the way to explore such novel 2D materials as economical alternatives to the expensive platinum-based catalysts for hydrogen generation.

## 2. Materials and Methods

### 2.1. Preparation and Characterization of Materials

SnSe films were deposited on mica substrates using the molecular beam epitaxy (MBE) technique, in which compound SnSe pieces with 5N purity (purchased from American Elements, Los Angeles, CA, USA) were used as the evaporation source and tin pellets with 5N purity (purchased from American Elements, Los Angeles, CA, USA) were employed as the compensation source, loaded in separate K-cells. The substrate temperature was kept at about 250 °C for film growth, while the SnSe source was heated up to 450 °C for evaporation. The temperature of the tin source varied in the range between 700 °C and 800 °C to compensate the Sn vacancies and regulate the electrical property of the SnSe film.

The crystal phase of the prepared SnSe films was examined by XRD, which was performed using a Rigaku Smartlab 9 kW X-ray diffractometer with the incident wavelength of 1.5406 Å (Rigaku Corporation, Tokyo, Japan). Atomic force microscopy (AFM) was employed to depict the film surface morphology using a Bruker NanoScope 8 (Billerica, MA, USA) in the tapping mode. Ultraviolet photoelectron spectroscopy (UPS) was used to determine the work function of the prepared SnSe film, which was recorded on an EscaLab 250 spectrometer (Thermo Fisher Scientific, Waltham, MA, USA) with an energy step of 20 meV, using He(I) radiation (hν = 21.22 eV) as the UV source. A Bio-Rad 5500 Hall system (Hercules, CA, USA) equipped with a permanent magnet with a magnetic flux density of 0.32 T was used to determine the electrical property applying the four-probe van der Pauw method.

### 2.2. Computation Details

Density functional theory (DFT) computations were performed using the plane-wave basis set in the VASP with the projector augment wave (PAW) method [28,29]. Exchange and correlation effects for the structural relaxation were approximated by generalized gradient approximation (GGA) utilizing the Perdew–Burke–Ernzerhof (PBE) functional [30,31]. The Grimme custom method for DFT-D3 correction was employed to precisely depict the impacts of van der Waals interactions [32,33]. The HSE06 (Heyd–Scuseria–Ernzerhof) functional was utilized for electronic structure computations because the PBE functional typically underestimates the bandgap value [28]. The cutoff energy was set to be 500 eV for the plane-wave basis set. The Brillouin zone (BZ) was sampled using a 8 × 7 × 3 k-point Monkhorst–Pack sampling grid for the bulk SnSe and a 2 × 2 × 1 grid for the SnSe monolayer. The convergence criteria of energy and force were 1 × 10^−5^ eV and 0.01 eV/Å, respectively. A vacuum layer of 15 Å was added along the c-axis of the SnSe monolayer to avoid the impact of the periodic layer.

**Figure 1:**
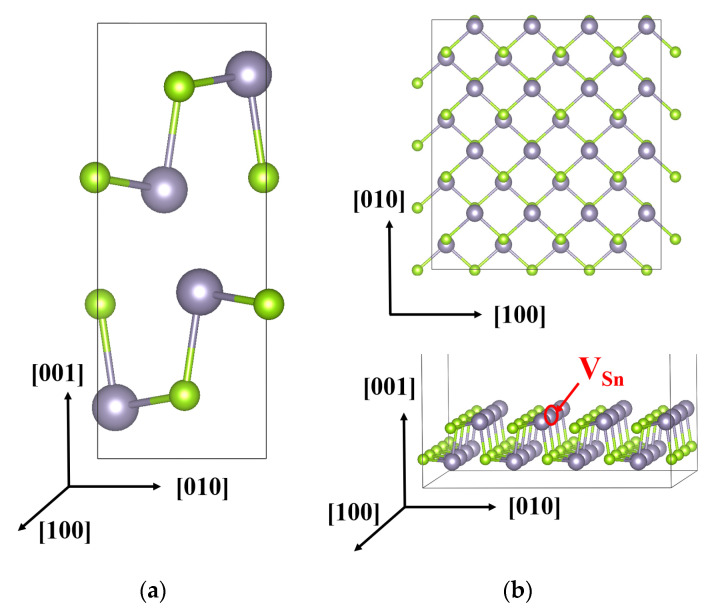
The structure of SnSe. (**a**) Unit cell of bulk SnSe. (**b**) Top view and perspective view of the constructed SnSe supercell. Navy-colored balls represent Sn atoms and green balls represent Se atoms. The red circle indicates an Sn vacancy.

To obtain further insights into the HER performance of SnSe, DFT simulations were conducted to compute the free energy (ΔG_H*_) of H adsorption, which is usually employed as a key indicator for HER activity. To find which surface is more conducive to the HER, an SnSe monolayer surface and a surface with one Sn defect introduced were constructed as shown in Figure 1.

The adsorption of H atoms on the surface was studied, and the hydrogen chemisorption energy was computed as follows:

(1) $$\Delta E_{H^{*}}=E_{slab+H}-E_{slab,clean}-\frac{1}{2}E_{H_{2},gas}$$

where $E_{slab+H}$ stands for the total energy of the adsorbed hydrogen atom on the surface, $E_{slab,clean}$ is the calculated energy of a clean surface, and $E_{H_{2},gas}$ is the total energy of an *H*_2_ molecule in the gaseous state.

The free energy of the systems can be expressed as follows:

(2) $$\Delta G_{H^{*}}=\Delta E_{H^{*}}+\Delta E_{ZPE}-T\Delta S_{H}$$

where $\Delta G_{H^{*}}$, $\Delta E_{H^{*}}$, $\Delta E_{ZPE}$, and $\Delta S_{H}$ denote the free energy of the system, the aforementioned adsorption energy, the zero-point energy change, and the entropy change between adsorbed hydrogen and hydrogen in the gaseous state at standard conditions, respectively; $\Delta S_{H}$ is roughly equal to $\frac{1}{2}\Delta S_{H_{2}}$, where $\Delta S_{H_{2}}$ is the entropy of an isolated H_2_ molecule in the gaseous state at standard conditions; therefore, the value of $T\Delta S_{H}$ is approximately −0.2 eV. $\Delta E_{ZPE}$ can be described as follows:

(3) $$\Delta E_{ZPE}=E_{ZPE}^{H}-\frac{1}{2}E_{ZPE}^{H_{2}}$$

where $E_{ZPE}^{H}$ and $E_{ZPE}^{H_{2}}$ represent the zero-point energy of an adsorbed hydrogen atom as well as of a hydrogen molecule in the gaseous state, respectively.

## 3. Results and Discussion

**Figure 2:**
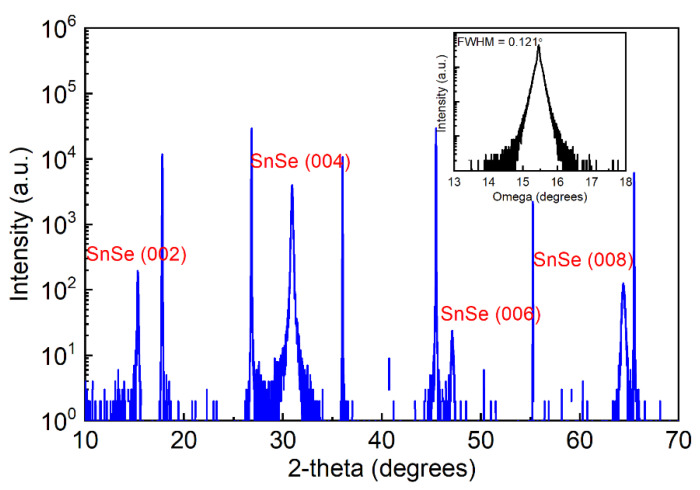
X-ray diffraction θ–2θ scan probing the out-of-plane orientation of the SnSe film on a mica substrate. The inset shows the rocking curve conducted on the SnSe (004) plane.

Being a 2D layered material, high-quality SnSe layers can be obtained on mica substrates, which also belong to the class of 2D materials providing a chemically inert, atomically flat, and electrically insulating surface [26]. As shown in Figure 2, only the diffraction peaks at 15.3°, 30.9°, 47.1°, and 64.4° originating from the SnSe (002) family planes emerge in the XRD pattern, suggesting a highly textured growth along its c-axis, namely perfect layer-by-layer stacking. In addition, an in-plane phi scan of the SnSe (016) plane with respect to the SnSe (001) plane was conducted by tilting the sample at an angle of χ = 24.71° and setting the incident x-ray angle of 2θ = 52.17° as shown in Figure S1 in the Supplementary Materials. As a result, the lattice constants of SnSe can be derived to be a = 4.42 Å, b = 4.19 Å, c = 11.57 Å. The XRD pattern and the calculated crystal parameters are in excellent agreement with the JCPDS database (No. 1089–0236). The inset in Figure 2 describes the XRD rocking curve carried out on the SnSe (004) plane with respect to a mica substrate. The narrow peak demonstrates an FWHM as small as 0.121°, which might be the narrowest value ever reported and indicates excellent quality of SnSe crystallinity [26,27].

**Figure 3:**
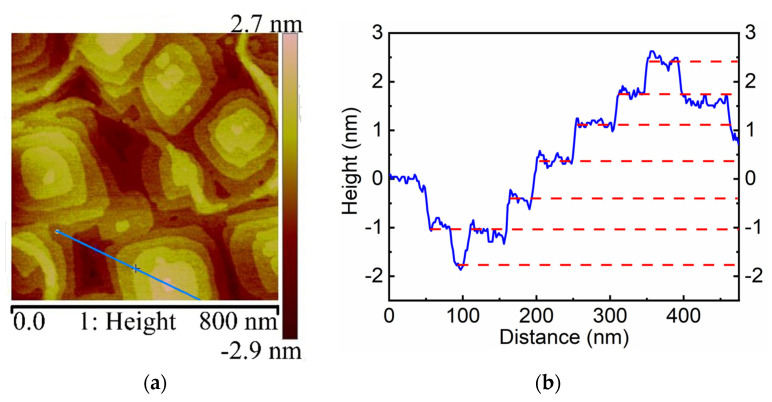
(**a**) AFM morphology of SnSe on a mica substrate. (**b**) The height profile along the blue line in (**a**).

The AFM morphology is displayed in Figure 3. The root-mean-square (RMS) roughness was calculated to be 1.03 nm (on a two-micron scale), indicating a very flat SnSe (001) surface. Orthorhombic terrace-like features emerged with the size of ~500 nm and a height of 0.68 nm on the average, signifying the monolayer thickness, which is consistent with the value obtained from the XRD data in Figure 2.

The Hall measurements revealed that the prepared SnSe film exhibited a p-type conductivity due to Sn deficiency during the crystallization, introducing acceptor states in the film. The Hall mobility was measured to be 34 cm^2^/(V·S) at the sheet resistivity of 1.5 × 10^4^ Ω/square. Fortunately, Sn defects can be effectively compensated by adding elemental tin atoms simultaneously during film growth and the amount of compensating tin atoms incorporated in the film can be precisely regulated by varying the elemental tin source temperature. As shown in Figure S2 in the Supplementary Materials, sheet resistivity can be adjusted three orders of magnitude larger than that without Sn compensation, making the SnSe crystal nearly perfect with the least Sn vacant defects.

**Figure 4:**
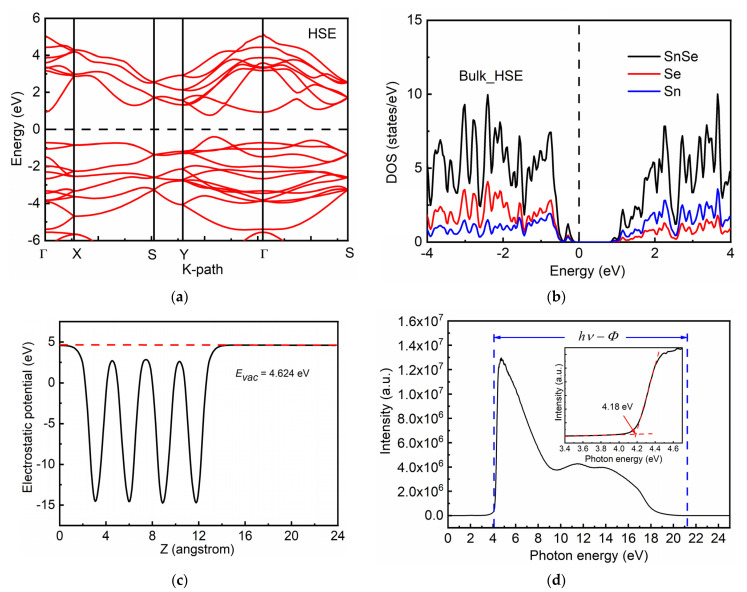
Energy diagrams of SnSe. (**a**) Energy bands in the HSE functional calculations. (**b**) DOS in the HSE functional calculations. (**c**) Average electrostatic potential of the SnSe (001) surface. The red dashed line shows the energy at the vacuum level. (**d**) Work function of SnSe measured by UPS.

First-principle calculations revealed that Sn vacancies in the film played an important role in electrocatalysis acting as reactive sites. Geometry optimization of the SnSe unit cell leading to the abovementioned lattice constants was executed before the simulations. The band structures and density of states (DOS) calculated using the HSE06 method for bulk SnSe are plotted in Figure 4a,b. It can be seen that the band nature of bulk SnSe is indirect and the band gap is computed as 1.20 eV, which agrees with our experimental value of 1.18 eV. To further investigate the electronic structure of the system, the work function of the SnSe (001) surface was simulated as well. The larger the work function, the less likely our system would lose electrons and the more stable it would be. The calculation formula of work function is as follows:

Φ = *E_vac_* − *E_F_* (4)

where Φ is the electronic work function, *E_vac_* is the energy of the vacuum level, and *E_F_* is the energy of the Fermi level. Through calculations, it was found that the energy of the vacuum level was 4.624 eV and the Fermi level was located 0.4563 eV above the valence band maximum. The average electrostatic potential is presented in Figure 4c. The work function of the SnSe (001) surface was thus calculated to be 4.1677 eV, which agrees well with the value of 4.18 eV derived from the UPS measurement as shown in Figure 4d.

**Figure 5:**
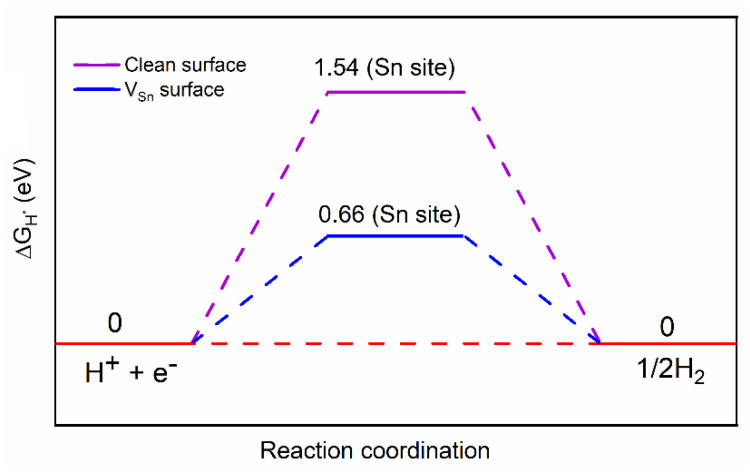
The adsorption Gibbs free energy of H adsorbed on the SnSe monolayer surface. Clean represents the clean SnSe monolayer surface. V_Sn_ represents the SnSe monolayer surface with one Sn defect.

HER activity was evaluated by plotting a two-state HER free energy diagram [31], which contains the initial H^+^ + e^–^ state, the intermediate adsorbed H* and the final ½H_2_ product. It is well-known that an optimum HER site has a free energy change $\Delta G_{H^{*}}$ of hydrogen adsorption close to zero [34,35]. The HER performance of the SnSe monolayer is summarized in Figure 5. It is clearly observed that the clean basal surface of the SnSe monolayer possesses a ΔG_H*_ value of 1.54 eV, demonstrating relatively poor HER activity. However, when an Sn defect site was introduced, it was obviously found that $\Delta G_{H^{*}}$ substantially decreased to 0.66 eV, namely by a factor of 2.3, indicating that the HER performance of SnSe can be greatly boosted by introduction of Sn vacancies acting as reactive sites.

**Figure 6:**
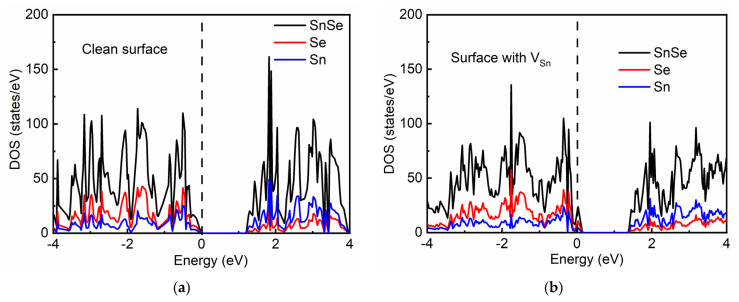
Density of states of two different surfaces. (**a**) Clean SnSe monolayer surface. (**b**) SnSe monolayer surface with one Sn defect.

To understand the effect of Sn vacancies on the electronic structure of the SnSe monolayer, the density of states was again computed with the presence of an Sn vacancy. Additional electronic states near the Fermi level appeared within the bandgap of SnSe as shown in Figure 6. Thus, the electrical conduction of V_sn_ was substantially enhanced, suggesting that electrons can easily transfer to the reactive sites on the surface, which is beneficial for continuous hydrogen generation. Therefore, DFT calculations demonstrated that Sn vacancies increased the electrical conductivity and reduced the $\Delta G_{H^{*}}$ value, leading to a great enhancement in the HER activity.

## 4. Conclusions

Hydrogen is regarded to be one of the most promising strategies for the development of clean and renewable energy, especially pushed by the carbon neutrality pledges from companies and governments around the world. For clean energy conversion, 2D materials have attracted much attention due to their unique structural and electronic properties, among which SnSe has been recognized as an economical alternative to expensive platinum-based catalysts for hydrogen evolution. In this work, SnSe layers with excellent crystallinity were prepared on a mica substrate using the MBE technique. The films exhibited a perfect layered structure and p-type conductivity, which were attributed to Sn vacancies. However, Sn defects can be easily and accurately regulated by a separate elemental Sn source in a wide range to meet the application requirements. First-principle calculations via the VASP revealed that it is because of the Sn defects more reactive sites are introduced, substantially lowering the Gibbs free energy of H adsorption on the SnSe surface and boosting the HER activity. Enhanced hydrogen evolution performance through controllable defect engineering demonstrated that such 2D SnSe shows great promise for hydrogen generation applications.

## Data Availability

Not applicable.

## Supplementary Materials

The following are available online. Figure S1: XRD in-plane phi scan of SnSe (016) with respect to the SnSe (001) plane; Figure S2: Variation of SnSe sheet resistivity with the tin compensation source temperature.
