# Supplementary material for: Fabrication of Highly Textured 2D SnSe Layers with Tunable Electronic Properties for Hydrogen Evolution
Source: Molecules. 2021 Jun 1;26(11):3319. doi: 10.3390/molecules26113319 (PMC8199299; doi:10.3390/molecules26113319)
Supplement: Supplementary file 1 [file molecules-26-03319-s001.zip › molecules-1237440-supplementary.pdf]

# Fabrication of Highly Textured 2D SnSe Layers with Tunable Electronic Properties for Hydrogen Evolution

**Qianyu Zhou [1,2,3,#], Mengya Wang [1,2,3,#], Yong Li [1,2,3], Yanfang Liu [1,2,4], Yuanfu Chen [2,4,*], Qi Wu [1,2,3,*] and Shifeng Wang [1,2,3,*]**

[1] Department of Physics, Innovation center of Materials for Energy and Environment Technologies, College of Science, Tibet University, Lhasa 850000, China

[2] Institute of Oxygen Supply, Center of Tibetan Studies (Everest Research Institute), Tibet University, Lhasa 850000, China

[3] Key Laboratory of Cosmic Rays (Tibet University), Ministry of Education, Lhasa 850000, China

[4] School of Electronic Science and Engineering, and State Key Laboratory of Electronic Thin Films and Integrated Devices, University of Electronic Science and Technology of China, Chengdu 610054, PR China

* To whom correspondence should be addressed: Yuanfu Chen (yfchen@uestc.edu.cn), Qi Wu (email: wuqi@utibet.edu.cn) and Shifeng Wang (email: wsf365@163.com),

[#] The authors contributed equally to this work.

## Supplementary Materials

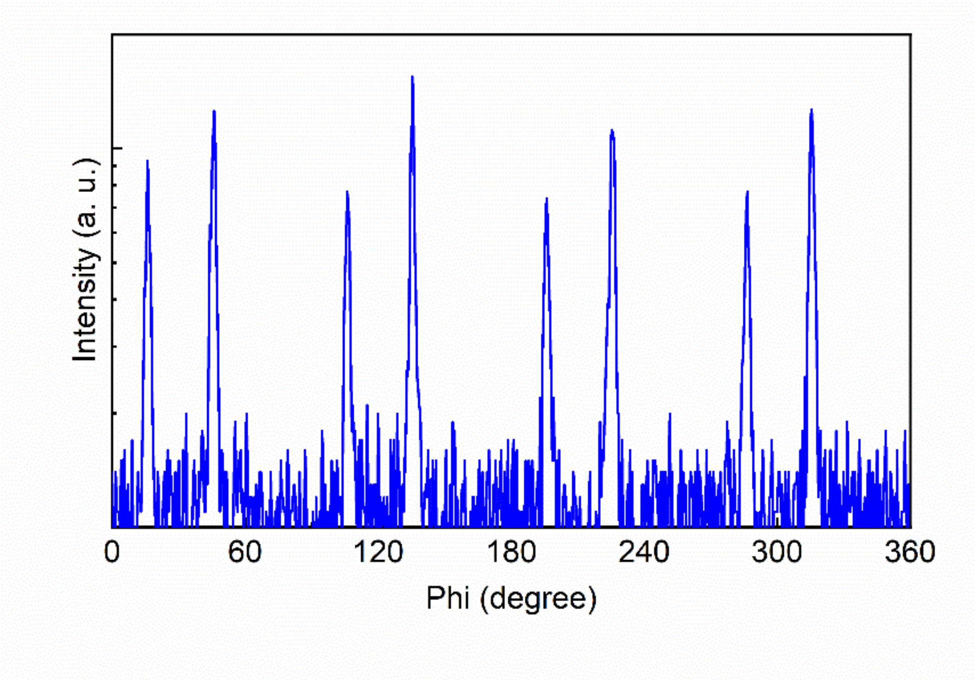


**Figure S1.** XRD in-plane phi scan of SnSe (016) with respect to SnSe (001) plane.

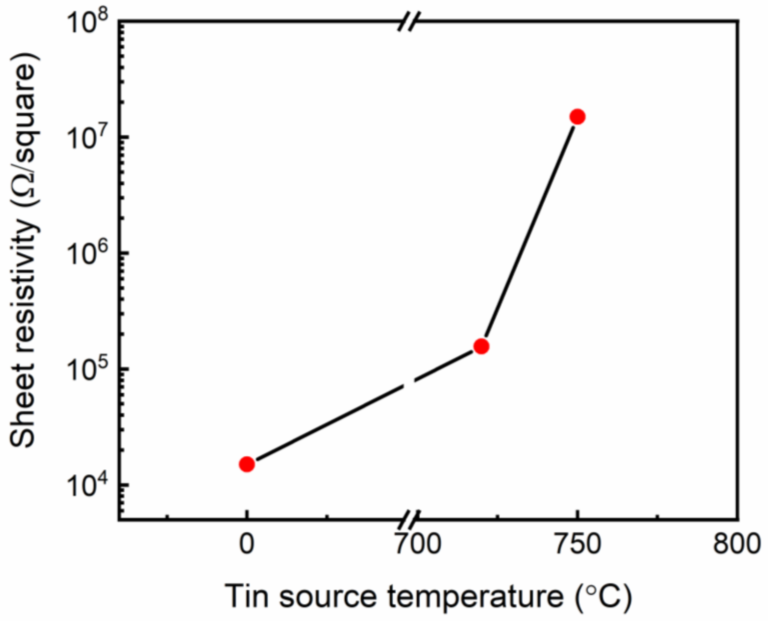


**Figure S2.** Variation of SnSe sheet resistivity with Tin compensation source temperature.
